# Co-Immunization With CHIKV VLP and DNA Vaccines Induces a Promising Humoral Response in Mice

**DOI:** 10.3389/fimmu.2021.655743

**Published:** 2021-03-24

**Authors:** Zhimin Zhao, Yao Deng, Peihua Niu, Jingdong Song, Wen Wang, Yongping Du, Baoying Huang, Wenling Wang, Leiliang Zhang, Ping Zhao, Wenjie Tan

**Affiliations:** ^1^National Health Commission (NHC) Key Laboratory of Medical Virology, National Institute for Viral Disease Control and Prevention, China CDC, Beijing, China; ^2^Key Laboratory of Laboratory Medicine, Ministry of Education, School of Laboratory Medicine and Life Sciences, Wenzhou Medical University, Zhejiang, China; ^3^Institute of Basic Medicine, Shandong First Medical University & Shandong Academy of Medical Sciences, Jinan, China; ^4^Shanghai Key Laboratory of Biomedical Protection, Department of Biomedical Protection, Faculty of Naval Medicine, Navy Medical University, Shanghai, China

**Keywords:** immunity, virus-like particle, DNA vaccine, chikungunya virus, co-immunization

## Abstract

Chikungunya fever is an acute infectious disease that is mediated by the mosquito-transmitted chikungunya virus (CHIKV), for which no licensed vaccines are currently available. Here, we explored several immunization protocols and investigated their immunity and protective effects in mice, with DNA- and virus-like particle (VLP)- vaccines, both alone and in combination. Both DNA and VLP vaccine candidates were developed and characterized, which express CHIKV structural genes (C-E3-E2-6K-E1). Mice were immunized twice, with different protocols, followed by immunological detection and CHIKV Ross challenge. The highest antigen-specific IgG and neutralizing activity were induced by DNA and VLP co-immunization, while the highest cellular immunity was induced by DNA vaccination alone. Although all vaccine groups could protect mice from lethal CHIKV challenge, demonstrated as reduced viral load in various tissues, without weight loss, mice co-immunized with DNA and VLP exhibited the mildest histopathological changes and lowest International Harmonization of Nomenclature and Diagnostic Criteria (INHAND) scores, in comparison to mice with either DNA or VLP vaccination alone. We concluded that co-immunization with DNA and VLP is a promising strategy to inducing better protective immunity against CHIKV infection.

## Introduction

Chikungunya fever (CHIKF) is a recurrent infectious disease caused by the chikungunya virus (CHIKV), which belongs to the genus Alphavirus of the Togaviridae family (1, 2). First documented in Tanzania in 1952, this virus has caused several CHIKF outbreaks, mainly in Africa and Asia. Widespread outbreak of CHIKF in recent years has made it a global public health problem (3).

CHIKV is a positive-stranded RNA virus that encodes six structural (C-E3-E2-6K/TF-E1) and four non-structural (nsP1, helicase nsP2, nsP3, and polymerase nsP4) proteins (4). The two alphavirus envelope glycoproteins E1 and E2, each containing a single transmembrane domain, are responsible for mediating viral attachment (E2) and membrane fusion (E1) (5, 6). The prefusion E1/E2 heterodimer is arranged in 80 trimeric spikes, resulting in a viral particle with an icosahedral structure (7, 8).

Currently, there are no licensed CHIKV vaccines available for use; however, potential vaccine candidates are classified into seven types: inactivated vaccines, subunit vaccines, live-attenuated vaccines, recombinant virus-vectored vaccines, virus-like particle vaccines, chimeric vaccines, and nucleic acid vaccines (9, 10). Virus-like particles (VLPs) are generated by expression of the CHIKV structural cassette from a DNA expression plasmid transfected into human cells. The expressed structural proteins form particles that are indistinguishable from intact virions, but are replication-incompetent because they lack genomic viral RNA. Recently, a VLP vaccine, expressing by the measles virus vector, entered phase 2 clinical trials (11, 12). While this vaccine has demonstrated good safety and tolerability, it must be administered at least twice to reach 100% seroconversion and induce the production of sufficient levels of neutralizing antibodies. Importantly, an antibody-dependent enhanced infection (ADE) was reported during the infection process of CHIKV (13), and the severity of CHIKV infection increased in the presence of a subdominant immune response after immunization in mice (14, 15). Therefore, the impact of ADE on vaccination should also be considered during vaccine design.

Previous studies have shown that both CHIKV VLP and DNA vaccines can elicit specific immunity and protect mice from a lethal CHIKV threat (16, 17). Recently, a study demonstrated that co-immunization with different vaccine candidates could improve adaptive immunity (18). Co-immunization with both West Nile DNA and inactivated vaccines provide a synergistic increase in immunogenicity of mice (19). In addition, co-immunization with virus-like particles and DNA vaccines induce protection against respiratory syncytial virus infection and bronchiolitis (20). To date, a limited number of studies have investigated the efficacy of CHIKV vaccines, specifically the development of immunity by using DNA and VLP vaccines alone, or in combination. This study provides a comprehensive analysis of the immunogenicity and efficacy of CHIKV DNA and VLP vaccines, administered both individually and in combination, in mice.

## Methods

### Cell Lines and Viruses

African green monkey Vero cells and human embryonic kidney 293T cells were grown in Dulbecco’s modified Eagle’s medium (DMEM) (Hyclone, South Logan, UT, USA), supplemented with 10% fetal bovine serum (FBS) (Gibco, NY, USA) and 1% penicillin-streptomycin (Gibco, NY, USA). The live attenuated vaccine, CHIKV 181/clone25 strain (Asian), was obtained from Terence Dermody through Add gene (pSinRep5-181/25ic, plasmid 60078) (21, 22). The CHIKV Ross infectious clone was provided by Dr. Ping Zhao (23). CHIKV 181/clone25 and CHIKV Ross infectious clone viruses were propagated and tittered in Vero cells.

### Construction of DNA-Based CHIKV Vaccine

**Figure 1:**
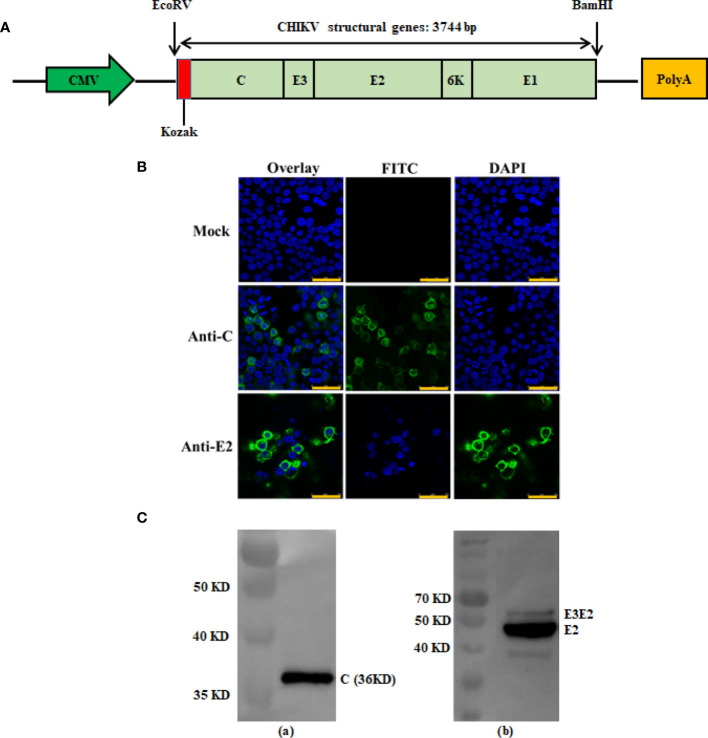
Construction and antigen expression analysis of recombinant DNA-based CHIKV vaccine candidate. Schematic diagrams of the recombinant DNA-based vaccine encoding CHIKV structural genes. The flanking enzyme sites used for cloning, Kozak expression element and CHIKV structural genes (C-E3-E2-6K-E1) are indicated and were cloned into the VRC-8301 vector **(A)**. Immunofluorescence staining images showing the 293T cells transiently transfected with the pVRC-CHIKV or VRC-8301 (mock) at 24 h post-transfection. The capsid and E2 protein (FITC) and cell nuclei (DAPI) were stained in green and blue, respectively. Scale bars, 50 µm **(B)**. Expression of pVRC-CHIKV constructs was confirmed *in vitro* using CHIKV Capsid and E2 rabbit polyclonal antibodies for the Western blot of CHIKV Capsid (left) and E2 (right) proteins expressed in 293T cells by Western blotting **(C)**.

The full-length gene encoding CHIKV structural poly protein (C-E3-E2-6K-E1) was amplified by polymerase chain reaction from CHIKV 181/25 with the following primers: CHIKV-forw 5’-ATCGCCACCATGGAGTTTATCCCAACCC-3’ and CHIKV-rev 5’-CGGGATCCTTAGTGCCTGCTAAACGAC-3’. The products of the CHIKV structural genes were digested with EcoRV and BamHI, and cloned into the expression plasmid VRC-8301, under the control of the cytomegalovirus (CMV) immediate-early gene promoter (provided by Dr. Gary Nabel, NIH) (24). The final expression construct was named pVRC-CHIKV (DNA-based CHIKV vaccine) and screened using PCR and double restriction enzyme digestion. It was confirmed *via* sequencing in both directions to ensure fidelity (**Figure 1A**).

### Indirect Immunofluorescence Assay

Briefly, 293T cells were transfected with pVRC-CHIKV and the VRC-8301 empty vector (mock)using PEI (Polyethylenimine) for 24 h, followed by fixing with pre-cooled 4% paraformaldehyde, mobilizing in 0.2% Triton X-100, and blocking in 10% goat serum in PBS. Subsequently, the cells were incubated with rabbit polyclonal antibodies (Alpha Diagnostic Intl Inc, USA) against the CHIKV Capsid and E2 proteins, at 37°C for 2 h. After washing with PBS, the cells were stained with secondary antibodies (FITC-labeled goat anti-rabbit IgG) and 0.1% DAPI at 37°C for 1 h. Images of cells were acquired using a Leica TCS SP8 confocal microscope with LAS software (Leica Biosystems, Wetzlar, Germany)

### Preparation and Purification of CHIK VLPs

**Figure 2:**
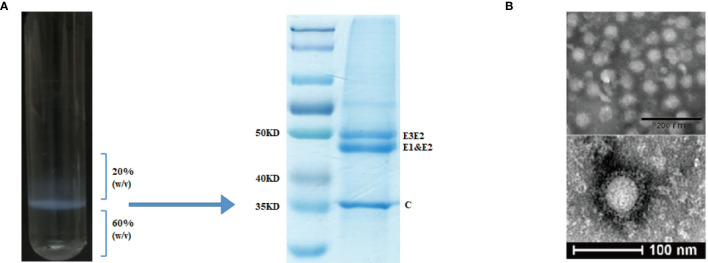
Purification and characterization of CHIK-VLPs. CHIK-VLPs separated at the 20-60% sucrose density gradient interface, SDS-PAGE followed by Coomassie blue staining **(A)**. Electron microscopy of negatively stained CHIK-VLPs purified by sucrose gradient **(B)**.

For the preparation of CHIK VLPs, sub-confluent 293T cell cultures in 225 cm^2^ tissue culture flasks were transfected with 45 μg of pVRC-CHIKV by using PEI. At 72 h post transfection, the culture media was collected, centrifuged at 4,000 rpm for 30 min at 4°C, and filtered through a 0.22 μm filter to remove cell debris. The VLPs were pelleted through a 20% sucrose cushion at 24,000 rpm for 4 h at 4°C, using a Beckman SW32 rotor. Pellets were re-suspended in TNE buffer containing 100 mM NaCl, 50 mM Tris-HCl, pH 7.2, 1 mM EDTA (Sigma, USA) and loaded on a discontinuous 60% and 20% sucrose gradient in TNE. Sucrose gradients were centrifuged at 24,000 rpm for 4 h at 4°C using a Beckman SW41 rotor. The VLPs at the interface of 20-60% sucrose were collected, diluted, and pelleted through a 20% sucrose cushion at 24,000 rpm for 4 h at 4°C. The pellets were suspended in TNE buffer and stored at -80°C, until further use. The VLPs were analyzed by SDS-PAGE for the presence of CHIKV structural polyprotein (**Figure 2**). VLPs were quantified based on specific E2 protein content, which was determined by SDS-PAGE and calculated using ImageJ software.

### Transmission Electron Microscopy

The purified CHIK-VLPs were negatively stained with 1% phosphotungstic acid (Ph 6.8). Briefly, 5 μL of the VLPs were placed on a carbon-Formvar coated copper grid for 1 min. Then grid was washed thrice in sterile triple-distilled water by floating the grid on water droplets for 45 s, to remove excess sample and sucrose. Finally, the samples were stained with PTA solution for 1 min. The air-dried grid was examined using a Tecnai12 transmission electron microscope (FEI, Eindhoven, Netherlands) at 120 kV and recorded with a CCD camera.

### Immunizations and Challenge

Female C57BL/6 mice, aged 6 to 7 weeks, were purchased from the Beijing Vital River Laboratory Animal Technology. All experiments were approved by the Committee on the Ethics of Animal Experiments of the Chinese Center for Disease Control and Prevention (China CDC).

**Figure 3:**
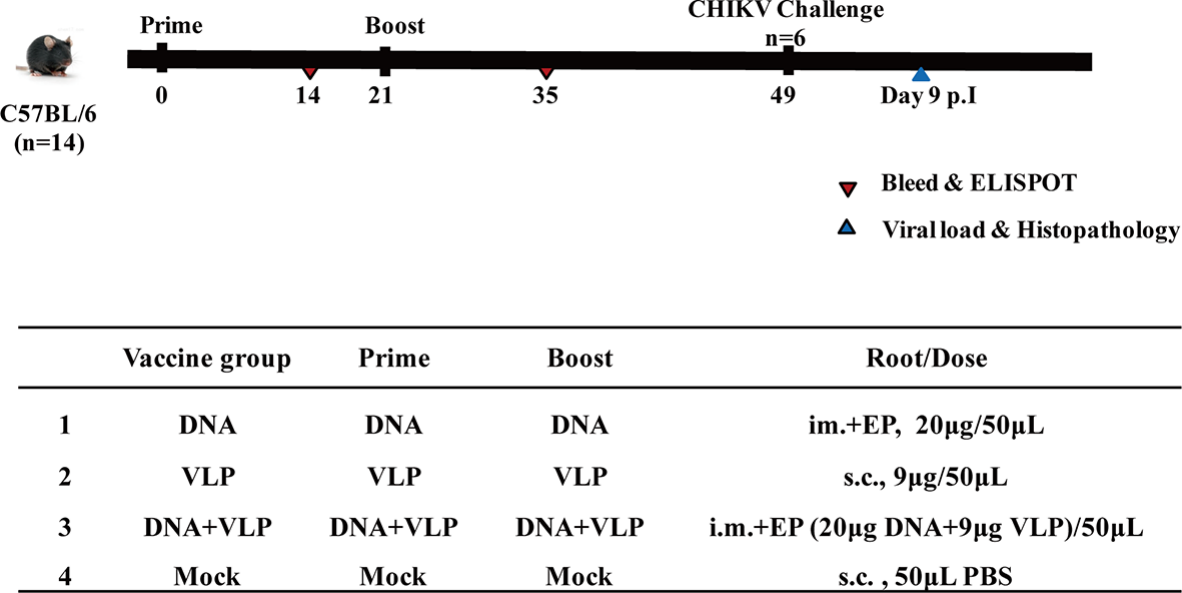
Immunization and challenging schema of CHIKV vaccines. Mice (n=14 per group) were either mock immunized with PBS or vaccinated with DNA, VLP or DNA+VLP. Blood (n=10) was collected for humoral immune response detection at day14 and 35 by ELISA and plaque reduction neutralization assays at day 35. 14 days after the prime and boost immunization, mice (n=4) were sacrificed and cellular immune responses were analyzed in splenocytes. Mice (n=6) were challenged with 1.7×10^7^ TCID_50_ of the CHIKV Ross by intranasal infection at day 49. Body weight, survival rate, tissue viral load, and histopathological changes were evaluated.

Mice were immunized (**Figure 3**) with DNA alone or co-immunized with DNA and VLP (hereafter DNA&VLP), on day 0 and day 21, *via* intramuscular injection plus electroporation (i.m.+Ep), and with VLP alone or the mock vector, by subcutaneous injection. Samples were taken for immunological detection on days 14 and 35, post-vaccination.

Mice were challenged with 1.7×10^7^ TCID_50_ of the CHIKV Ross, which belongs to the East Central South Africa lineage (ECSA), in a total volume of 50 μL by intranasal infection. The body weight and survival rate of mice were monitored daily. Animals were sacrificed either 14 days post-infection, or earlier if weight loss of more than 20% was observed. The heart, liver, spleen, lung, kidney, brain, and hind limbs of mice were harvested after sacrifice (3 mice per group). Half of the tissues were used for the determination of viral load, and the other half were fixed in a 4% formalin solution and sent to Beijing Zhongkewanbang Biotechnology Co., Ltd., for the preparation of hematoxylin and eosin (H&E) stained sections, and pathological evaluation.

### Enzyme-Linked Immunosorbent Assay

96-well plates (Thermo Fisher Scientific, USA) were coated with E1 (50 ng/well), E2 (50 ng/well), and VLPs (100 ng/well), followed by blocking with 200 μL of 10% goat serum in PBS at 37°C for 2 h. The plates were washed five times with PBST, followed by incubation with serially diluted post-vaccinated sera, including mock sera, at 37°C for 1 h, followed by five washes with PBST. HRP-labeled goat anti-mouse IgG [1:5000], IgG1 [1:1500], and IgG2c [1:1500] antibodies were added at 37°C for 1 h. After washing (as described above), 100 μL of TMB was added to each well, incubated for 2 min, and then quenched with 50 μL of 2M H_2_SO_4_. Optical density values were measured using SPECTR Ostar Nano (BIO-GENE, China) at a wavelength of 450 nm. The IgG2c/IgG1 ratios were calculated to define the T cell phenotype induced by vaccination since IgG2c and IgG1 levels are indicative of Th1 and Th2 responses, respectively (17).

### Neutralizing Antibodies

Sera were heat-inactivated for 30 min at 56°C, diluted 2-fold from a starting dilution of 1:40, and mixed with an equal volume (10–15 pfu/well) of CHIKV Ross for 1 h at 37°C. Virus dilutions without sera were prepared as controls. After 1 h of incubation, virus dilutions were applied to Vero cell monolayers for 1 h at 37°C, and then overlaid with 4% methylcellulose. Plates were incubated for 3 days at 37°C and 5% CO_2_. The overlay was then removed, and monolayers were stained with 1% crystal violet. Plaques were counted, and the neutralization titer was defined as the highest dilution resulting in a 50% reduction in plaque number (24, 25).

### IFN-γ ELISpot Assay

ELISpot assays were performed as previously reported (26). Briefly, 96-well ELISpot plates were coated with a purified anti-mouse IFN-γ capture antibody (BD ELISPOT Set, USA) and incubated overnight at 4°C. The following day, 6×10^6^ splenocyte suspensions from each group were stimulated with 8 μg/μL of either C (ACLVGDKVM), E1 (HSMTNAVTI), E2 (IILYYYELY) dominant single peptides (27, 28), or Con A (positive control), in the presence of a positive control. The plates were then incubated at 37°C in 5% CO_2_ for 20–24 h. The next steps were performed according to the manufacturer’s instructions (BD ELISPOT Set, USA). A spot forming unit (SFU) was used to represent a T cell-secreting IFN-γ. The plates were then detected using an ELISpot plate reader (Biosys, So. Pasadena, CA).

### Determination of Viral Load

To quantify the relative amount of viral RNA in the respective tissues, viral RNA was extracted from the heart, liver, spleen, lung, kidney, and brain samples using the automated Magna Pure method and a Total Nucleic Acid Kit (Roche Diagnostics), following the manufacturer’s instructions. CHIKV RNA was detected with specific TaqMan probes by using one-step RT-PCR (Master RNA hybridization probes, Roche), performed on a Chromo 4 machine (Bio-Rad). The primers and probes used for CHIKV RNA quantification were CHIKV-forw AAGCTCCGCGTCCTTTACCAAG; CHIKV-rev CCAAATTGTCCTGGTCTTCCT; and Probe: Fam-CCAATGTCTTCAGCCTGGACACCTTT-BHQ1 (29). For absolute quantification, standard curves were generated using 10-fold dilutions of CHIKV Ross RNA templates of known concentration (tittered in Vero cells by TCID_50_). Based on repeated standard curves, the formula was obtained: y=-3.641x+31.76 (y means CT, x means index). The extracted viral RNA from tissues were used to obtain the CT number using qRT-PCR. Weight of each tissue was measured prior to extraction. From these known data, the results were calculated and expressed as TCID_50_ per gram of tissue (30).

### Statistical Analysis

The treatment groups were compared by two-way analysis of variance (ANOVA) and Tukey multiple comparison tests (31). Data are shown as the mean ± the standard error of the mean (SEM). Additional data were analyzed using GraphPad Prism version 7 (GraphPad Software LLC). A P value of <0.05 was considered significant.

## Results

### Characterization of DNA and VLP Vaccines

To verify the expression of the CHIKV structural polyprotein, a western blot and an indirect immune fluorescence assay of pVRC-CHIKV transfected cells were performed using rabbit anti-CHIKV Capsid (C) and E2 antibodies, which demonstrated the expression of the capsid protein and envelope E2, as expected (**Figures 1B, C****)**. No expression was detected in mock-transfected cells.

CHIK VLPs were produced by pVRC-CHIKV transfected 293T cells and purified by 20-60% discontinuous sucrose density gradients (**Figure 2A**). The purified particles were analyzed by SDS-PAGE, in which E1 and E2 glycoproteins migrated together and formed a single band at ~47.3 kDa, while the precursor E3E2, was observed as a higher molecular weight band at approximately ~54.6 kDa. The capsid protein formed a distinct band at ~36 kDa (**Figure 2B**). Electron microscopy was used to further examine the formation of CHIK VLPs. The observed VLPs had diameters of about ~65-70 nm, showing morphological characteristics similar to those of standard CHIK VLPs (**Figure 2C**) (7, 16).

### Co-Immunization With DNA and VLP Elicits Highest Humoral Immunity

**Figure 4:**
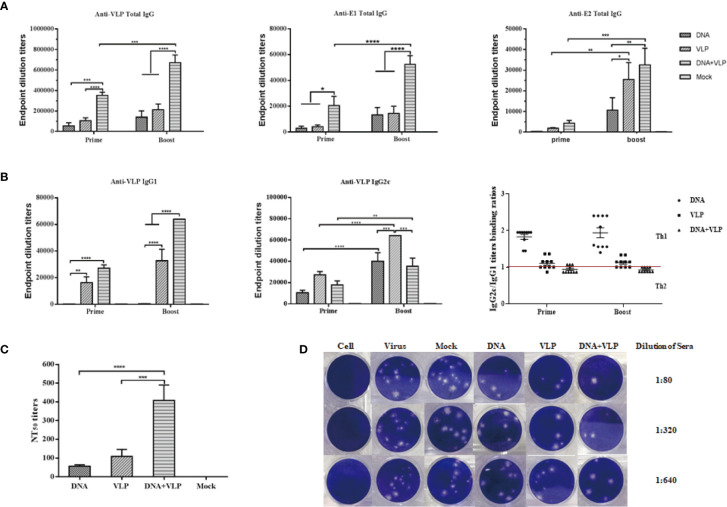
Co-immunization-induced broad CHIKV antigen-specific IgG antibody responses and high cross-neutralization antibody response in mice. Anti CHIKV VLP, E1 and E2 antibody measurement by ELISA. Sera were collected at day 14 and 35 after the prime and boost immunization and total IgG produced was measured in each group **(A)**. IgG subtype ELISA analysis. The graph shows VLP specific IgG1 and IgG2c antibody levels in mice, IgG2c/IgG1 binding ratios are shown for mice immunized with DNA, VLP or DNA+VLP **(B)**. Neutralizing antibody titers against CHIKV Ross were determined by plaque reduction neutralization assays at day 35 **(C)**. Representative results of the plaque reduction neutralization (PRNT) assay for the detection of neutralization activity in the sera of mice **(D)**. Approximately 10-15 pfu of the virus stock (CHIKV Ross) was used to infect Vero cells in 12-well plates with heat-inactivated sera from immunized mice 14 days after the boost immunization. PRNT_50_ was calculated after the plaques were counted. Data are from one experiment of three independent experiments and presented as the mean ± SEM (n=10). The statistical analysis among groups analyzed by two-way analysis of variance (ANOVA) and Tukey multiple comparison tests, ****p<0.0001, ***p<0.001, **p<0.01, *p<0.05.

In this study, we tested whether co-immunization with CHIKV VLP and DNA vaccines would increase the immunogenicity of either VLP or DNA immunization alone, in mice. It was found that co-immunization using DNA and VLP vaccines induced significantly stronger VLP-, E1-, and E2- specific antibody responses, in comparison to DNA or VLP immunization alone (p<0.001 or p<0.0001, respectively) (**Figures 4A–C**). Mice in the DNA&VLP group clearly produced robust VLP- and E1-specific antibodies after the first injection (p<0.001), which was higher than those of mice in the DNA or VLP only group (p<0.0.001). When comparing the specific titers of mice against E2 proteins in each immunized group, it was found that the highest levels of anti-E2 antibodies were observed in the DNA&VLP group, after boost immunization, followed by the VLP only and DNA only groups (**Figure 4B**). From the previous study results, we know that IgG1 and IgG2c isotypes were the main IgG subclasses induced after DNA or VLP immunization (7, 17). To further characterize the immune response generated, VLP-specific antibody subtypes were characterized. Co-immunization with DNA and VLP could induce both IgG1 and IgG2c antibodies, with significantly higher IgG1 antibody levels in comparison to IgG2c antibody levels, while the DNA or VLP only immunization groups mainly induced IgG2c antibodies (**Figure 4B**).

Neutralizing antibodies play an important role in the process of CHIKV infection and viral clearance. In comparison to the mock group, all immunized groups were observed to induce neutralizing antibodies in mice after the booster injection, which could cross-neutralize the heterologous CHIKV Ross strain. Among them, the DNA or VLP only immunized group induced lower neutralizing antibody titers, while co-immunization significantly increased neutralizing antibody titers (**Figures 4C, D****)**. Taken together, co-immunization induced higher levels of humoral immunity (IgG and neutralizing antibodies) than either DNA or VLP immunization alone.

### Co-Immunization With DNA and VLP Suppresses T Cell-Mediated Inflammation Responses in Mice

**Figure 5:**
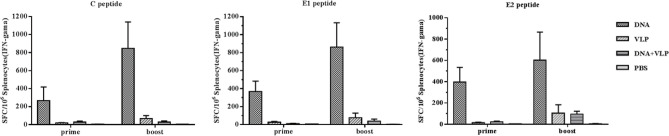
Enzyme-linked immunospot assays of IFN-γ secretion in immunized mice. Splenocytes were isolated from mice (n=4) and stimulated with CHIKV C, E1, and E2 peptides at day 14 and 35 after the prime and boost immunization. Splenocytes secreting IFN-γ were quantified using ELISpot assays. The data represent the mean ± standard error [SEM], with units of SFCs per million splenocytes.

To better understand immunity induced by different immunization protocols, we determined the induction of IFN-γ-producing C-, E1-, and E2-specific T cells in the spleens of mice after the vaccination. Upon *in vitro* stimulation of spleen cells with C, E1, and E2peptides, we measured IFN-γ-producing cell spots to evaluate the T cell response. We found that the DNA-immunized group induced the highest level of IFN-γ secretion after immunization. Co-immunization did not increase the induction of IFN-γ secretion which was found to be lower than that of either the DNA or VLP only groups (**Figure 5**). These results confirmed that the combination of DNA and VLP induced robust Th2-polarized VLP-specific immunity and suppressed T cell-mediated immunity in mice.

### Co-Immunization Can Protect Mice From Lethal CHIKV Threat

**Figure 6:**
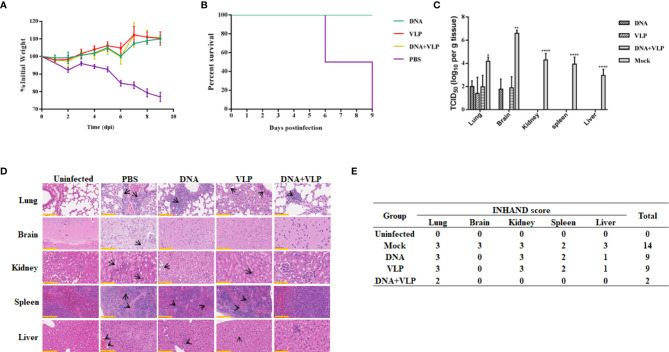
Co-immunization protects mice from lethal CHIKV challenge. Mice infected with 1.7×10^7^ TCID_50_ of the CHIKV Ross were monitored daily for body weight **(A)** and survival (mean ± standard error [SEM], n=6) **(B)**. Mice (n=3) sacrificed at day 9 post challenge. The heart, liver, spleen, lung, kidney, brain, and hind limbs of mice were harvested after sacrificed. Tissue supernatants were analyzed for viral load by real-time qRT-PCR. Data represent three independent experiments and are shown as the mean ± standard error [SEM]. The statistical analysis among groups analyzed by two-way ANOVA, ****p<0.0001, **p<0.01, *p<0.05. None viral load detected in heart and hind limbs of each group **(C)**. Histopathological analysis of tissues at day 9 after challenge. Section of different tissues were stained with hematoxylin and eosin. Scale bar, 100 μm. The black arrowhead stood for lesions in tissue **(D)** (3 mice per group and 1 section per tissue). INHAND scores of challenged mice organs, on a severity scale of 0–3 (none, mild, moderate, and severe) **(E)**.

To determine whether there was enhanced protection against CHIKV threat in DNA and VLP co-immunized mice, in comparison to groups immunized with DNA or VLP alone, immunized mice were challenged with 1.7×10^6^ TCID_50_ CHIKV Ross through intranasal infection 3 weeks after boost immunization. Body weight, survival rate, tissue viral load, and histopathological changes were evaluated (**Figure 6**). We found that none of the vaccinated mice suffered from disease or death (**Figure 6B**), and their body weight did not significantly decrease (**Figure 6A**). In contrast, mice in the control group started to gradually lose weight 3 days after challenge (**Figure 6A**), with hind limb paralysis or death occurring by the 6^th^ day, and all control mice died by the 9^th^ day (**Figure 6B**). Tissue viral load analysis revealed no viral nucleic acid traces in the heart, liver, spleen, and kidney tissues of the immunized groups. Only very low levels of viral RNA were detected in the lung and brain of the immunized groups, in comparison to the mock group (**Figure 6C**).

We performed a histological examination of mouse tissue obtained on the 9^th^ day, and the results of H&E staining are shown in **Figure 6D**. In lung tissue sections, it was found that mock and DNA-or VLP-immunized mice exhibited a widened alveolar septum, infiltrated blood vessels, bronchial inflammatory reaction, trocar formed around blood vessels, and a homogeneous powder was found in the bronchial and bronchiolar cavities. In contrast, mice in the DNA&VLP group showed only mild local alveolar space widening and a small amount of inflammatory cell infiltration (**Figure 6D**). Although viral nucleic acid was detected in the brain tissue of immunized mice, no obvious lesions were observed in brain tissue sections (**Figure 6D**). We observed severe lesions in kidney tissue, and minor lesions in spleen and liver tissue, of mice vaccinated with either DNA or VLP, while co-immunized mice did not show obvious lesions in kidney, spleen, and liver tissue sections. INHAND score analysis showed that the mock group had 14 points, the DNA or VLP group had 9 points, and the co-immunized group had only 2 points, which was significantly lower in comparison to that of the other groups (**Figure 6E**). Taken together, DNA and VLP co-immunization provided the best protection after CHIKV Ross challenge.

## Discussion

In this study, we developed novel CHIKV DNA and VLP vaccines, and evaluated their immunological and protective effects in mice, using different vaccination protocols. The results showed that all vaccination protocols were effective in protecting mice from lethal CHIKV challenge. Notably, co-immunization of mice with DNA and VLP vaccines exhibited the mildest histopathological changes and lowest INHAND scores, in comparison to mice with either DNA or VLP vaccination alone. This promising protection observed in mice co-immunized with DNA and VLP vaccines may be associated with higher levels of humoral immunity (IgG and neutralizing antibodies).

In a previous study, a DNA vaccine expressing E1-E2-C CHIKV proteins mainly induced IgG1 subtype antibodies, while a VLP vaccine induced a balanced IgG1/IgG2c antibody response after immunization (17); however, IgG2c subtype antibodies were mainly induced in our immunized groups. The difference in vaccine outcomes between this previous report and the present study may have resulted from differences in the target antigens (the CHIKV full structure gene was used in this study) and vaccination protocols were used. Of note, natural CHIKV infection is dominated by IgG2c (32). Furthermore, we found that the DNA&VLP group induced specific IgG antibodies that recognized VLP, E1, and E2, in mice after the first immunization, and the level of IgG antibodies significantly increased after booster immunization. Co-immunization can also induce high levels of IgG1 and IgG2c antibodies against VLP, in which the Th2 response, represented by the presence of IgG1 antibody, dominates. It has been found that mixing polypeptides, proteins, inactivated viruses, or VLPs with DNA can synergistically enhance humoral immune response, improve neutralizing antibody levels, and produce a lasting immune protective effect (33–35).

Here, PRNT assays were applied to evaluate the cross-neutralizing ability of immunized sera to CHIKV Ross. It was found that co-immunization induced the highest level of cross-neutralizing antibodies, which was significantly higher in comparison to mice treated with either DNA or VLP vaccines alone. This was consistent with a previous report where an HIV/SIV DNA vaccine, combined with protein in a co-immunization protocol, elicited the highest humoral responses to the viral envelope in mice and macaques (36). In addition, previous respiratory syncytial virus (RSV) vaccine studies have shown that adding DNA to VLP to immunize mice, could provide long-term protection against RSV infection, without the risk of lung disease (20).

In 2003, Wang et al. found that immunization with either a DNA vaccine or a protein vaccine could activate T cells, while the combination of DNA and protein inhibited T cell immunity (37). In combined HIV DNA and protein immunization protocols, the cellular immune response was enhanced, which led to an increase in IFN-γ, TNF-α and multi-functionality among CD8^+^ T cells and dominance of Th1-polarized Ab-specific antibodies (36). While DNA and protein combined immunization in Alzheimer’s disease induced robust Th2-polarized Ab-specific antibodies, suppressed or eliminated unwanted adverse T cell-mediated immune responses. At the same time, co-immunization reduced the production of inflammatory cytokine IFN-γ and increased the production of IL-4 in CD4^+^ T cells, suggesting that an anti-inflammatory effect was induced in the co-immunized mice (38). Here, we found that the cellular immune response, upon treatment with individual VLP or DNA vaccines, was stronger than that induced by co-immunization with CHIKV DNA and VLP. In addition, the combination of VLP and DNA failed to enhance the cellular immune response. Therefore, we conclude that co-immunization of CHIKV VLP with DNA vaccines inhibits cellular immunity, which is otherwise increased by either DNA vaccination alone.

When mice were challenged with a lethal dose of CHIKV Ross, we found that all immunized mice did not develop the disease or die. Further analysis of histopathological sections revealed that the mock group, as well as the VLP, or DNA immunization groups had significant lung lesions, while mice in the DNA&VLP group had only mild lung lesions. This may be related to the nasal inoculation of CHIKV Ross. CHIKV Ross exhibited a certain neurotoxicity (39). Previous studies have shown that infection with CHIKV Ross can damage brain tissue in mice (40). Here, only the mock group had significant brain lesions, while the brain tissues of immunized mice were normal. However, it was found that different degrees of pathological damage were observed in the kidney, spleen, and liver of mice with either VLP or DNA immunization, while co-immunization did not reveal any pathological changes in these tissues. We suggest that co-immunization has a better protective effect as compared to treatment with DNA or VLP vaccines alone, especially with respect to the production of neutralizing antibodies, which play a vital role in the process of CHIKV infection and viral clearance (41–43). Whether these differences in pathology due to humoral response are more effective in protection against CHIKV infection than the T cell mediated response remains to be elucidated. From our results, we found that the T cell-mediated response was significantly higher in the DNA only group than in the co-immunization group, but it did not provide better protection than co-immunization. No significant difference was found between the VLP only group and the co-immunization group T cell-mediated response, while co-immunization provided better protection than the VLP only group. This may be because co-immunization induced higher levels of humoral response than VLP or DNA immunization. We found that the humoral response is more effective in protection against CHIKV infection than the T cell-mediated response. Therefore, future CHIKV vaccines should focus on generating protective antibodies.

Several limitations of the current study need to be examined. CHIKV is an arthritogenic virus. Future studies will explore whether the DNA&VLP vaccine strategy could be an excellent strategy to reduce the joint footpad swelling and muscle pathology in the well-defined footpad model. Second, in the DNA only group, a T-cell mediated response was observed, which is very likely a Th1 response (high IgG2c, low IgG1). Additional studies should identify the role of these T cells. Lastly, we only observed the DNA&VLP vaccine strategy in C57BL/6 mice. Future studies should upgrade the vaccine production and explore protection efficacy and related mechanisms in larger animals.

In summary, our data revealed that co-immunization with DNA and VLP vaccines did not enhance the cellular immune response in mice, but significantly enhanced humoral immunity and induced higher levels of antibodies with cross-neutralizing activity, in comparison to treatment with either component alone. After CHIKV Ross challenge, co-immunization provided greater cross-protection in mice. We concluded that the VLP and DNA co-immunization method developed here, might be a very promising strategy to improve immunity and protection against CHIKV infection.

## Data Availability Statement

The original contributions presented in the study are included in the article/**Supplementary Material**, further inquiries can be directed to the corresponding author.

## Ethics Statement

The animal study was reviewed and approved by Committee on the Ethics of Animal Experiments of the Chinese Center for Disease Control and Prevention.

## Author Contributions

Conceptualization: WT and ZZ. Formal analysis: ZZ, PN, and YD. Investigation: ZZ, JS, YPD, and WW. Resources: LZ and PZ. Writing—original draft preparation: ZZ and WT. Writing—review and editing: ZZ, LZ, and WT. Project administration: BH, WLW, and WT. All authors contributed to the article and approved the submitted version.

## Funding

This work was supported by the National Key Research and Development Program of China (2018YFC1200602 and 2016YFD0500301) and the National Major Project for Control and Prevention of Infectious Disease in China (2016ZX10004001-003 and 2018ZX10731-101). The funding agencies had no role in the study design, data collection, data analysis, decision to publish, or preparation of the manuscript.

## Conflict of Interest

The authors declare that the research was conducted in the absence of any commercial or financial relationships that could be construed as a potential conflict of interest.
